# A Scoping Review of Studies on Assistive Technology Interventions and Their Impact on Individuals with Autism Spectrum Disorder in Arab Countries

**DOI:** 10.3390/children10111828

**Published:** 2023-11-20

**Authors:** Maha Al-Hendawi, Esraa Hussein, Badriya Al Ghafri, Sefa Bulut

**Affiliations:** 1 Department of Psychological Sciences, College of Education, Qatar University, Doha P.O. Box 2713, Qatar; esraa.mohamed@qu.edu.qa (E.H.); badriya.alghafri96@outlook.com (B.A.G.); 2 Department of Counseling Psychology, School of Education, Ibn Haldun University, 34494 İstanbul, Turkey; sefa.bulut@ihu.edu.tr

**Keywords:** autism spectrum disorder, assistive technology, Arab countries, scoping review

## Abstract

The rising prevalence of autism spectrum disorder (ASD) in Arab countries necessitates evidence-based interventions. Assistive technology (AT) presents a promising approach. However, data on the pervasiveness of AT use and its effectiveness for individuals with ASD, specifically within Arab countries, remain scarce. Objective: To review the current literature on the AT interventions and outcomes reported for individuals with ASD in Arab countries. Methods: A scoping review adhering to PRISMA guidelines was undertaken to explore the utilization of AT, segmented into three categories: low-technology (low-tech), mid-technology (mid-tech), and high-technology (high-tech) devices. Results: Twelve studies had a pooled sample of 1547 participants, primarily male school-aged children with ASD. The AT applications evaluated ranged from low-tech visual schedules and support to high-tech virtual reality systems. Studies have reported the potential benefits of AT in improving communication, social, academic, adaptive, and functional abilities; however, comparative evidence between AT interventions is limited. The identified barriers to the adoption of AT included caregiver uncertainty about the use of AT and a lack of awareness of AT among professionals and the Arab community in general. Conclusion: Available studies suggest that the adoption of AT can enhance the skills of individuals with ASD in Arab countries. However, more rigorous studies across diverse demographic groups and Arab national regions are needed to strengthen the evidence base and provide appropriate recommendations.

## 1. Introduction

Autism spectrum disorder (ASD) is a neurodevelopmental disorder characterized by persistent social interactions and communication challenges, accompanied by repetitive behaviors that manifest in the early developmental period [1,2,3,4]. The complex and heterogeneous symptoms and behaviors associated with ASD require an early and accurate diagnosis, as well as the timely implementation of evidence-based interventions to improve developmental outcomes. This is particularly critical in regions with limited healthcare resources and public awareness of ASD.

Assistive technology (AT) encompasses a wide range of low-tech and high-tech aids, including visual support, social narratives, speech-generating devices, video modeling, and mobile technologies [5]. AT has been shown to be effective in supporting children with ASD in various settings and developmental domains [6,7,8]. Studies have shown that AT can facilitate engagement, learning, communication, and the inclusion of children with ASD in home, school, and community environments [9,10,11]. International conventions affirm access to appropriate AT as a human right for individuals with disabilities. A growing body of evidence indicates that AT can enhance functional skills critical to independence and participation in children with ASD. For example, research shows that communication aids, such as picture exchange systems, can improve language abilities, reduce sensory issues, and build social interaction skills [12,13,14].

The prevalence of ASD has increased worldwide and this trend is mirrored in the Arab world [12,13,14]. Salhia et al. (2014) examined the epidemiological landscape of ASD in Arab Gulf countries, revealing a prevalence range of 1.4 to 29 cases per 10,000 individuals and identifying potential metabolic, autoimmune, and environmental risk factors [15]. In Qatar, a prevalence of 1.14% was reported among school-aged children between 2015 and 2018 [2]. Furthermore, the World Population Review reported a high prevalence of ASD in Arab countries such as Qatar (151.2/10,000 or 1 in 66), the United Arab Emirates (UAE) (1 in 89), Oman (1 in 93), Bahrain (1 in 97), and Saudi Arabia (1 in 99), highlighting the need for early diagnosis and effective intervention [16].

The increase in the prevalence of ASD has led to a greater focus on interventions and the use of AT [8,17,18]. The effective implementation of interventions such as AT is often intertwined with the sociocultural and infrastructure dynamics of a region, emphasizing the importance of examining its adoption and results in Arab countries to accurately assess its impact [1,6,19,20]. Specifically, the recognized potential of AT to improve functional outcomes calls for an understanding of its impact within the Arab context, unveiling region-specific benefits and challenges, and assisting in devising effective implementation strategies. This aligns with the global recognition of access to AT as a human right, advocating the need to explore its prevalence and outcomes in diverse regions to foster global equity in access to AT and its benefits [8,17,18].

This review used a scoping framework to synthesize existing evidence on AT interventions for people with ASD in Arab countries [21]. Using this framework allows for a comprehensive synthesis of the available research evidence to determine the extent, range, and nature of evidence on the use of AT in ASD within the Arab sociocultural context. This scoping review methodology facilitated the resolution of the broad research questions underpinning this study.

1. What types of AT tools have been examined in studies conducted in Arab countries?
2. What outcomes have been reported when AT is used with people with ASD in Arab countries?
3. What factors influence AT adoption by people with ASD in Arab countries?

## 2. Methodology

### 2.1. Literature Search Strategy

A comprehensive literature search was conducted to examine the use of AT by individuals with ASD in Arab countries using a scoping review framework [21]. Electronic databases PubMed, ERIC, Education Source, Education Database, PsycArticles, Academic Search Ultimate, Psychology Database, and Taylor and Francis Online were systematically searched. The search was limited to articles published in English in peer-reviewed journals from 1 January 2013 to 31 December 2023.

The search strategy employed the keywords “Autism” and “Technology,” along with either “Arab” or “Middle East.” These keywords were derived following a meticulous examination of titles, abstracts, and terms within articles previously identified as pertinent and by evaluating the outcomes of exploratory searches.

The settings for AT interventions included in this review were categorized as specialized autism centers, educational settings, and diverse environments. Specialized autism centers provide customized interventions and education for individuals with ASD using evidence-based practices. Educational settings included mainstream and special education schools attended by students with ASD. Diverse settings refer to various other environments, such as homes, community spaces, vocational sites, and clinics, where assistive technology use has been studied.

#### 2.1.1. Inclusion Criteria

The inclusion in this review was based on five distinct criteria: (a) articles must be in English and peer-reviewed, (b) publication dates between 2013 and 2023, (c) a focus on Arab individuals diagnosed with ASD, (d) involvement of AT tools, and (e) studies conducted in diverse settings, including schools, homes, and other relevant environments. Exclusion criteria encompassed studies published in books, book chapters, and conference papers.

#### 2.1.2. Screening and Study Selection

The abstract of each identified article was independently screened by two reviewers and marked as Yes (Y) or No (N) in the corresponding columns of the spreadsheet, indicating whether it met each of the five predetermined inclusion criteria. Only articles for which both reviewers marked Yes for all inclusion criteria were reviewed and analyzed in the full text. For abstracts lacking sufficient information to determine eligibility, the two reviewers briefly reviewed the full text to reach a consensus on whether the inclusion criteria were met. Inter-rater reliability was established by having the two reviewers independently code a random 30% sample (n = 559) of the abstracts initially selected, achieving a high agreement rate of 94%. Any coding discrepancies were resolved through discussions between the reviewers until a consensus was reached. This rigorous systematic screening ensured that the included studies accurately represented the target population, interventions, comparators, and outcomes.

#### 2.1.3. Definition and Types of AT

AT refers to any device, equipment, or system that aims to maintain or improve the functional capabilities of people with disabilities. AT supports skill development and enhances opportunities for children with disabilities through interventions, training, and technical guidance [22]. AT is commonly classified as low-, mid-, or high-tech based on its features and training needs [23]. Low-tech devices are non-electronic, simple, and customizable aids such as visual schedules, sensory tools, and adapted utensils (Table 1). They require minimal training to use and are inexpensive [24,25]. Mid-tech refers to basic electronic devices such as audio recorders, screen readers, and switch-adapted toys, bridging low-tech and advanced options. High-tech devices are sophisticated electronic technologies such as speech-generating devices, virtual reality platforms, and robotic systems. They enable personalized support but can be complex and costly [26].

#### 2.1.4. Assessment of Risk of Bias

Selection bias was evaluated in the reviewed studies based on the sampling methods and group comparability. The widespread use of convenience sampling in most studies indicated a high risk of selection bias. Performance bias was gauged by examining the blinding of participants and researchers; however, the lack of reported blinding in most studies indicated a high risk in this area. Detection bias was considered based on whether the outcome assessors were blinded or not. However, many studies did not detail the blinding procedures, suggesting a possible risk of detection bias. Attrition bias was determined by examining the completeness of the outcome data, with most studies showing low dropout rates, indicating a low risk of attrition bias. Reporting bias was assessed in terms of the selective reporting of results. The absence of preregistered protocols in many studies suggested a potential risk of reporting bias.

#### 2.1.5. Data Extraction and Synthesis

A structured data extraction process was developed to systematically review and synthesize the relevant information from the included articles. A customized Excel worksheet was created to capture key data points, with columns for the type of AT, publication year, study design, outcomes evaluated, country, setting, and age and gender of the participant. A descriptive analysis of the extracted data was performed using STATA statistical software version 18 to synthesize the results of all the studies and summarize the evidence. Frequencies and percentages were calculated for categorical variables such as type of AT, country, setting, and demographics of the participants. For continuous variables, means and standard deviations or medians and ranges were calculated as appropriate.

## 3. Results

### 3.1. Study Selection

**Figure 1:**
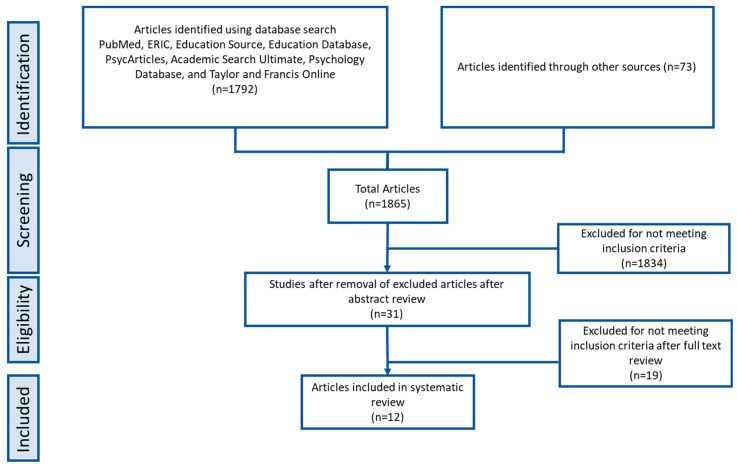
Prisma flow chart.

Initial database searches identified 1865 records after removing duplicates (Figure 1). Two independent reviewers screened the titles and abstracts of these records against predefined eligibility criteria. A total of 1834 records were excluded because they were irrelevant because they did not focus on individuals with ASD (n = 1456), did not investigate AT (n = 256), or were not conducted in Arab countries (n = 122). The full-text articles of the remaining 31 records were retrieved and formally assessed for eligibility by two reviewers. Of these 31 articles, 19 were excluded for the following reasons: not being peer-reviewed journal articles (n = 5), published in languages other than English (n = 3), not assessing AT interventions (n = 6), had outcomes unrelated to AT use (n = 3), and included children without ASD diagnoses (n = 2). After these screening stages, 12 studies satisfied all the inclusion criteria and were included in the qualitative synthesis for this review. In total, 12 studies met the eligibility criteria and were included in this scoping review (Table 2).

The bias assessment of the 12 included studies indicated a high risk of selection bias in 11 studies (91.7%) stemming from the predominant use of convenience sampling methods without comparability between groups (Table 3). All studies (12 studies, 100%) also had a high risk of performance bias because blinding of participants and researchers was not commonly reported. Detection bias was unclear in 1 study (8%) and high in 5 studies (41.7%) as blinding of outcome assessors was frequently not described. However, all studies (12 studies, 100%) had a low risk of attrition bias, with reasonably complete outcome data and minimal dropouts. Finally, all studies had an unclear (1 study, 8%) or high risk of reporting bias as preregistered protocols were rarely used and selective reporting of results could not be ruled out.

### 3.2. Key Pooled Findings

The included studies had sample sizes ranging from 3 to 1168 participants, comprising mainly children but some adults up to 26 years of age (Table 2). There was a greater representation of male participants than female participants in most studies where gender was reported. Various study designs were used, including single-case experiments [29,33,34], surveys [22,27], randomized controlled trials [32], and qualitative methods [36]. Studies examined a range of ATs, from low-tech adaptations to high-tech virtual reality systems. Studies indicated the potential benefits of AT in improving abilities in areas such as communication [22,32], social skills [29,34], academic performance [36], and cultural knowledge [33]. Studies also provided information on caregiver and teacher awareness and perspectives on technological use [22,27].

### 3.3. Regional and Type Distribution

**Figure 2:**
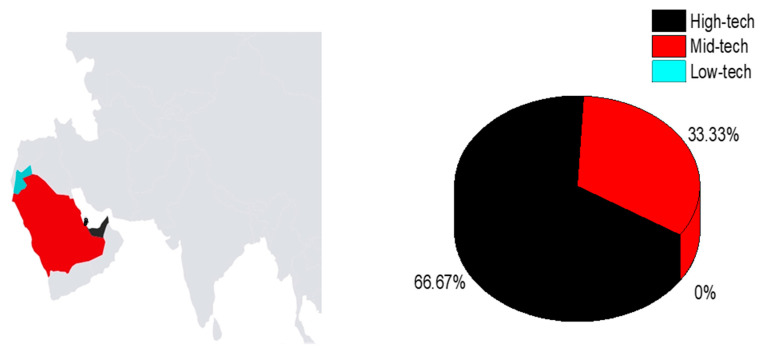
Geographical distribution of studies in the Arab region and Distribution of AT types (N = 11).

Most studies were carried out in the Gulf countries, including seven from the UAE [29,30,31,32,33,34,35,36], one from Qatar [27], and two from Saudi Arabia (KSA) [1,28]. One such study was conducted in Jordan [36]. One study included researchers from Qatar, the UAE, and the USA [37]. Figure 2A illustrates the geographical distribution of the studies within the Arab region and Figure 2B presents the types of AT used in these studies.

### 3.4. Research Settings

The studies examined were categorized into three distinct settings: specialized autism centers, educational settings, and diverse settings.

In the first category, specialized autism, three and four studies were conducted in controlled environments that were overlooked by experts [30,32,36]. For example, Fteiha (2017) conducted a study at the Dubai Autism Center, where participants were divided into three groups [32]. The first experimental group engaged with the CompuThera program to enhance reading proficiency, the second group engaged with the Language Master program to improve reading abilities, and the control group received conventional language training. The second category, educational settings, encompassed four studies conducted in school environments that promoted a sense of familiarity and comfort among participants [27,29,33,35]. A notable aspect of this setting was the designated resource room used for assessments, which ensured a consistent environment. Assessments were performed by qualified educators to maintain methodological rigor.

The third category, diverse settings, included studies conducted in various settings. For instance, Safi et al. (2021) [34] conducted their study in participants’ homes [28] and Borgestig et al. (2021) [31] conducted their study in both school and home settings [31]. Cabibihan et al. focused on different advanced ATs from the perspective of sensing technologies [35]. Additionally, some studies, such as Alsari et al. (2020) [28] and Alabbas and Miller (2019) [22], utilized virtual environments through online platforms and social media to engage a broader research sample, including community members, healthcare professionals, and augmentative and alternative communication (AAC) users [22,37].

### 3.5. Participant Characteristics

Most studies showed a higher number of male participants than female participants, as observed in previous studies [22,29,30,33,34]. The age range of the participants varied widely, with the youngest being 1 year old in the study by Alabbas and Miller (2019) [22] and the oldest being 26 in the study by Borgestig et al. (2021) [31]. One study included participants aged ≥18 years without specifying an upper age limit [38].

### 3.6. Sensory Technology and Outcome

**Figure 3:**
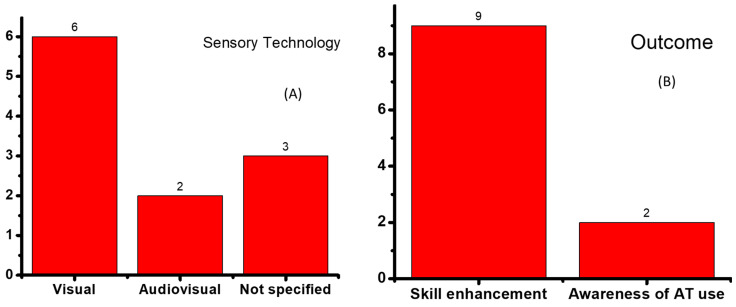
(**A**) Type of AT (N = 11). (**B**) Outcome (N = 11).

An analysis of the selected articles revealed distinctions in terms of sensory technology, specifically visual and audiovisual technology (Figure 3A). Six studies focused on visual technology [28,30,31,33,34,35]. Two studies focused on audiovisual technology [28,36]. Four studies [22,27,32,37] did not specify a specific AT category.

The outcomes were organized into two groups (Figure 3B): skill enhancement and awareness of AT utilization. Nine studies focused on enhancing various skills [22,30,31,32,33,34,35,36]. One study did not focus on specific outcomes [37]. Two studies [27,37] investigated awareness of the importance of AT for children with autism.

### 3.7. Technology Stage

These studies discussed the development and utilization of various technologies (Table 1 and Table 2). Studies of children with ASD and technology covered different stages, including technologies as potential products, products in development, and products in active use. The technological descriptions varied based on the research focus, ranging from detailed accounts of individual applications to specific technological solutions and more generic descriptions, resulting in a relatively general categorization of the types of technologies used.

### 3.8. Aims of Employing AT

The analysis of the selected studies revealed diverse objectives for utilizing AT in individuals with ASD. These purposes broadly fell into three major categories: exploring caregivers’ and teachers’ perspectives and awareness, diagnostic and early intervention strategies, and initiatives for skill enhancement.

Three distinct studies were identified in the first category: Alabbas and Miller (2019 [22]), Al-Attiyah et al. (2020 [27]), and Alsari et al. (2020 [28]), who utilized surveys to delve into the perceptions and awareness of caregivers and teachers regarding AT. These studies employed surveys to effectively address their research inquiries. Alabbas and Miller (2019) explored Saudi Arabian caregivers’ perceptions by surveying the problems they encountered with typical routines, the solutions they found to the problems, the AT they used in the solutions, and their training and feelings of competence in using AT. Al-Attiyah et al. (2020 [27]) utilized a survey to explore teachers’ perceptions about integrating AT into educational settings for children with disabilities. Alsari et al. (2020 [28]) developed and distributed a survey to obtain information on awareness, accessibility, and funding for AAC services and devices within the Kingdom of Saudi Arabia (KSA).

The second category included three studies that focused on diagnosis, with Siyam and Abdallah (2022 [35]) aiming for earlier diagnosis through mobile technology. Siyam and Abdallah (2022 [35]) focused on earlier diagnoses and investigated the use of mobile technology for the coordination of therapy and learning for students with disabilities. The third category encompassed ten studies on using AT to enhance abilities in individuals with ASD, including communication, social, academic, and daily living skills. For example, Alzyoudi et al. (2015 [29]) evaluated video modeling to improve social skills development, whereas Borgestig et al. (2021 [31]) and Fteiha (2017 [32]) investigated AT interventions to enhance communication abilities. Safi et al. (2021 [34]) explored virtual voice assistants for improving speech and social interaction skills. Sweidan et al. (2019) [36] developed a smartphone application to teach linguistic, mathematical, and social skills to Arabic-speaking children with ASD. Cabibihan et al. focused on sensing technologies in general [39]. Banire et al. (2015) [30] pursued developing a customized learning system framework to guide the creation of software tailored to autistic learners’ needs. Olsen et al. (2018) [33] utilized video modeling to teach culture-specific dressing skills to participants with ASD.

### 3.9. Types of AT

The reviewed studies employed a diverse range of AT tools categorized as mid-tech and high-tech interventions. Notably, high-tech devices were the most prominent in the Gulf region, with eight studies utilizing advanced technologies [28,30,31,34]. For example, in Saudi Arabia, Alsari et al. (2020) [28] distributed surveys via social media and email to collect AAC device usage data [37]. Given the high prevalence of communication disorders in the country, AAC’s nontechnological and high-tech tools for nonverbal communication are particularly crucial. Similarly, in the UAE, Alzyoudi et al. (2015) [29] used television video modeling as a high-tech social skills intervention for children with ASD [29]. Banire et al. (2015) developed a computer-based learning system to teach Quran recitation while sustaining attention [30]. In a multicenter study, Borgestig et al. (2021) used eye-gaze-controlled computers to enhance communication and engagement [31]. Expanding beyond the Gulf, Sweidan et al. (2019) created a smartphone app in Jordan to teach linguistic, mathematical, and social skills through interactive games and activities [36]. Siyam and Abdallah (2022) investigated mobile technology to coordinate educational plans and underscore participatory design principles in the UAE [35]. Other reviewed studies focused on interventions such as video modeling for teaching culture-specific skills [33], virtual voice assistants for improving speech and social abilities [28], and computer programs for building language competencies [32].

### 3.10. Effectiveness of Using AT

Several studies demonstrated the potential of AT in enhancing social skills in individuals with ASD [29,31,34,36]. Virtual voice assistants improved vocabulary, phrase production, and social interaction compared to traditional therapies in three children [34]. An Android app was most effective for mild-to-moderate ASD when used for limited durations under supervision [36]. Eye gaze technology increased expressive communication in 17 participants with complex needs [31].

Two studies showed a positive impact of AT on cognitive and language skills. Hybrid visual learning systems increased attention span and on-task behaviors more than traditional teaching in children with ASD [30]. Language development software provided reinforcement and enhanced skills [32]. One study demonstrated that video modeling was an acceptable and effective method for teaching culture-specific dressing skills to Emirati children, which parents acknowledged as important [33]. Two surveys provided insights into stakeholder AT awareness and perspectives [27,28]. Caregivers frequently used technologies, but some experienced feelings of inadequacy in their use, in contrast to teachers who demonstrated high adoption proficiency and endorsed the benefits. Awareness of augmentative communication was higher among professionals than community members.

## 4. Discussion

This scoping review synthesized evidence from 12 studies on AT interventions for individuals with ASD in Arab countries. The key findings were that the AT tools were primarily aimed at communication, social, academic, and cultural skills. Most studies involved mid- to high-tech AT, likely reflecting the relatively advanced infrastructure in the Gulf countries where these studies were conducted. Only one randomized controlled trial demonstrated the efficacy of AT in improving language skills, and the majority of the evidence was derived from small observational studies. The reported outcomes included gains in communication, social interactions, academics, and cultural knowledge. High adoption was observed among teachers and caregivers, indicating an increasing mainstream acceptance of AT in Arab countries. However, disparities in awareness and cost and a lack of collaboration emerged as barriers to the widespread adoption of AT. These results are consistent with those of previous studies on the use of AT for people with intellectual disabilities and ASD [17,39].

### 4.1. Type of AT and Geographical Variation

The reviewed studies reflected a predominance of mid- and high-tech AT, primarily in Gulf countries. This prevalence can be attributed to the relatively advanced economic and technological infrastructure of the Gulf countries, which allows for greater investment in sophisticated AT solutions compared to other Arab countries with limited resources [19]. In low-income countries, the widespread availability of high-tech and mid-tech AT may be limited, especially for impoverished citizens [26]. In contrast, low-tech assistive devices tend to see a higher adoption owing to their cost-effectiveness, simpler mechanics, and minimal training requirements [24,40]. It should be noted that low-tech AT can be made by families without requiring specialist input; for example, homemade supports such as rolled towels for sitting aids and weighted utensils to facilitate feeding exemplify the adaptability of low-tech solutions [41]. Such ingenuity in using readily available materials can enhance a child’s engagement in natural family settings and surroundings. However, making a decisive remark on the efficacy of different types of ATs is challenging due to the absence of comparative evidence evaluating the differential efficacy of various types of AT within the Arab region. In particular, this lack of rigorous comparative research is not confined to the Arab region but is a global issue, indicating the urgent need for more extensive research on the comparative evaluation of a wide range of AT tools and technologies [17].

### 4.2. Impact of AT on Outcomes

The reported results included improved communication skills, social skills, academics, and cultural knowledge. Our analysis found only one randomized controlled trial on the use of AT for ASD [32]. It involved 12 children and found that AT effectively improved language skills in autism. As noted above, previous research has also shown positive effects of AT on communication abilities, including mutual attention, verbal skills, imitation, and stereotypical reduction [17,39]. Specifically, a review by Syriopoulou-Delli and Gkiolnta analyzed 13 studies on AT in children with ASD and found positive immediate effects on communication skills such as mutual attention, verbal skills, and imitation, as well as reduced stereotypy [39]. Similarly, Maseri et al. analyzed 15 studies, revealing that autism-assistive apps improve verbal communication abilities in children with ASD [17]. Another key finding of our review was the association between AT use and improved social interaction in multiple studies. Technologies, such as virtual assistants and autism applications, promote social engagement and communication skills [28,34], confirming the results of other studies on the potential of AT to address ASD social communication challenges [5,7,17,18].

Importantly, AT affects cognitive and language skills more than cognitive skills alone. The CompuThera program [32], autism apps [36], and visual hybrid development learning systems [30] have improved these abilities, highlighting the adaptability and learning enablements of AT. A multicenter study demonstrated the benefits of eye-gaze-controlled computers [31], providing empirical evidence for AT and emphasizing multidisciplinary implementation. These positive outcomes demonstrate the versatility of AT and the potential for customized interventions that meet the unique learning needs of ASD. Overall, evidence indicates that thoughtfully designed technology tools aligned with areas of difficulty in autism spectrum disorder can create opportunities for greater social connection, relationship building, and participation. By supporting autistic learning styles and offering individualized scaffolding, assistive technology shows promise in improving real-world social interaction abilities and fostering greater involvement in children with autism spectrum disorder.

### 4.3. Adoption and Barriers

The high adoption among early intervention teachers [27] and caregiver use in daily routines [22] indicate a positive trajectory toward mainstream TA in educational and home settings. The use of mobile technology to coordinate educational plans [35] demonstrates the potential of AT to improve coordination and monitoring in inclusive classrooms, ensuring continuous assessment and adaptation to evolving student needs. Caregiver uncertainty, disparity between professional and public awareness, and low acceptance have emerged as critical barriers to the adoption of AT [1,22,28,42], reflecting the global challenges encountered in its implementation [17,38,43]. These findings imply that collaboration between speech therapists, occupational therapists, and other experts is integral to fully utilizing AT to address the diverse needs of patients with ASD. The high use of assistive technology among teachers [27] and caregivers [22] indicates the growing mainstream acceptance of these tools in Arab countries. This positive trajectory toward integrating assistive technology into standard educational and home settings for individuals with autism spectrum disorder can be further encouraged through comprehensive competency-building and training programs for stakeholders such as educators, therapists, and family members [1,22,28,44]. Addressing knowledge deficiencies and attitudinal barriers that impede wider assistive technology adoption through greater awareness and improved access to evidence of its benefits is also key to maximizing acceptance and uptake [1,22,28,44]. With dedicated efforts to build stakeholder capabilities and understanding of assistive technology, the mainstream integration of these beneficial tools into regular practice for individuals with autism spectrum disorder in Arab countries can be accelerated.

### 4.4. Age Diversity

Most studies focused on children, with minimal evidence of the effectiveness of AT across other age groups. However, ASD symptoms manifest differently across various developmental stages, resulting in changing support requirements over one’s lifespan [37]. For example, an assistive technology intervention focused on learning social skills may be suitable for a young child, whereas an adult may benefit more from technology aids in gaining employment or independent living skills. Examining tailored assistive technology strategies for youth, mature adults, and elderly people with autism spectrum disorder will offer insights into optimizing the benefits across age groups. Taking a lifespan developmental approach to study assistive technology interventions for individuals with autism spectrum disorder will be key to ensuring that these tools can improve outcomes and enhance functioning at all ages.

### 4.5. Knowledge Gaps and Implications for Future Research

The 12 included studies identified several key factors affecting the use of AT for ASD in the Arab region. However, significant knowledge gaps persist. More controlled clinical trials are critical to guide the appropriate and equitable adoption of the immense potential of AT to improve ASD outcomes in Arab countries and worldwide. Moreover, with regard to AT types, visual and audiovisual technologies have been the most studied, whereas research on other options of sensory approaches remains limited. This is despite the well-known sensory processing challenges present in populations with ASD, suggesting a mismatch between current AT priorities and user needs [37,44]. The diverse range of technologies adopted in these studies, from concepts to active tools, indicates the need to standardize development, evaluation, and implementation [45]. More research is necessary in naturalistic home and community settings where social inclusion is the goal [3]. These nurturing-regulated environments accommodate the unique requirements of students with ASD, thereby enhancing their potential and development [46]. Most research has focused on children. However, AT use lacks fixed age boundaries in ASD, likely owing to ASD’s varying manifestations across ages and phases, contributing to evolving needs [47]. Addressing age diversity will provide insights into tailoring AT to meet ASD demands across age groups. Addressing stakeholder competencies and attitudinal gaps may promote their adoption. Small samples and reliance on surveys rather than robust experiments also introduce bias. Addressing these limitations through rigorous, diverse, and extensive research is essential to firmly guide evidence-based practice.

### 4.6. Limitations

This review has several limitations that restrict the generalizability of the findings regarding the use of AT for ASD in Arab countries. The small sample sizes and reliance on surveys and observational studies rather than controlled experiments introduce a potential bias. Most studies focused on children, with limited evidence across age groups. There was also greater emphasis on visual and audio AT interventions than on other sensory modalities.

## 5. Conclusions and Future Directions

This scoping review underscores the potential benefits but provides limited evidence of AT for children with ASD in the Middle East. Preliminary studies have revealed promising outcomes for communication, academic, adaptive, and social skills. However, substantial gaps exist, including a lack of data on the perspectives of families and individuals with ASD and minimal research on low-tech solutions to improve accessibility. Rigorously designed studies that compare AT with standard practices are urgently required. Implementation research should identify optimized training, support, and capacity-building models for the sustainable use of AT in educational and clinical contexts. Policy and advocacy initiatives must address funding, infrastructure, and attitudinal barriers that inhibit access. This review highlights critical steps for strengthening the evidence base through expanded, rigorous research and simultaneous efforts to enhance equitable access and capacity surrounding AT. Key priorities include increasing stakeholder participation, improving methodological quality, and focusing on functional and participatory outcomes. Collaborative dedication among researchers, practitioners, policymakers, and the autism community is essential for realizing the full benefits of AT.

## Figures and Tables

**Table 1:** Classification of AT.

Category	Description	Examples	Cost	Training Needed	Benefits
Low-Tech	Simple non-electronic aids to enhance skills. Highly customizable.	Visual schedules, adapted utensils, sensory tools.	Low (under $50)	Minimal to none	Provides structure, makes tasks simpler.
Mid-Tech	Electronic devices/software to increase access to curricula. Require basic skills.	Audiobooks, adapted keyboards, speech-to-text.	Low to moderate ($100–$1000)	Basic device training often needed.	Allows greater independence for academic work.
High-Tech	Sophisticated electronic systems requiring extensive training and customization.	Speech-generating devices, virtual reality, robotics.	High (over $1000)	Extensive professional training required.	Provides personalized, intensive support tailored to individual needs.

Actual costs and features of devices may vary. Details provided are tentative.

**Table 2:** Summary of key characteristics of included studies.

Study	Country	Age and Sample Size	Intervention and Outcome Measures	Technology Used	Key Results
(Alabbas and Miller, 2019 [22])	Saudi Arabia	1–5 year; N = 41	Assistive technology; Daily routines; Qualitative	Online survey	High % of ASD and routine problems reported; AT most used for bathing and playing.
(Al-Attiyah et al., 2020 [27])	Qatar	N = 183	Assistive technologies; Teacher perceptions; Quantitative; Descriptive	Assistive technologies	High AT use by teachers.
(Alsari et al., 2020 [28])	Saudi Arabia	18 y or above; N = 1168	AAC services and devices; AAC awareness; Access, Funding; Quantitative	AAC high-tech devices	Significant difference in AAC awareness between groups.
(Alzyoudi et al., 2015 [29])	UAE	5–7 y; N = 5	Video modeling; Social skills; Qualitative; Single subject	TV, video	Effective for improving social skills.
(Banire et al., 2015 [30])	UAE	5–7 y; N = 11	Visual hybrid development learning system; Attention; Mixed methods	Visual hybrid Quran learning system	Increased attention with VHDLS.
(Borgestig et al., 2021 [31])	Sweden, UAE, USA	3–26 y; N = 17	Eye-gaze controlled computer; Communication; Functional independence; Quantitative	EGCC	Increased communication and functional independence.
(Fteiha, 2016 [32])	UAE	8–12 y; N = 12	AT computer programs; Language skills; Quantitative; Single subject	CompuThera	Greater language gains pre- to post-test.
(Olsen et al., 2018 [33])	UAE	7–15 y; N = 3	Video modeling; Dressing skills; Qualitative	Computer	Improved dressing skills.
(Safi et al., 2021 [34])	UAE	4–11 y; N = 3	Virtual voice assistants; Speech; Social interaction; Qualitative; Single case	Apple Siri	Positive effects on speech and social interaction.
(Siyam and Abdallah, 2022 [35])	UAE	6–10 y; N = 4	Mobile technology; IEP coordination; Qualitative; Participatory design	Mobile app (IEP-Connect)	Good usability and satisfaction.
(Sweidan et al., 2019 [36])	Jordan	5–13 y; N = 100	Android app; Language; Math; Social skills; Quantitative	Android app	Most improvement in Level 1; noticeable skill improvement.
(Cabibihan et al., 2017 [37])	UAE, Qatar, USA	-	Sensing technologies; ASD screening and intervention; Qualitative	Sensing technologies	Room for improvement remains in reliability and usability.

**Table 3:** Assessment of risk of bias in included studies.

Study	Selection Bias	Performance Bias	Detection Bias	Attrition Bias	Reporting Bias
Alabbas and Miller (2019 [22])	High	High	Unclear	Low	Unclear
Al-Attiyah et al. (2020 [27])	High	High	High	Low	Low
Alsari et al. (2020 [28])	High	High	Low	Low	Low
Alzyoudi et al. (2015 [29])	High	High	High	Low	Low
Banire et al. (2015 [30])	High	High	Low	Low	Low
Borgestig et al. (2021 [31])	Low	High	Low	Low	Low
Fteiha (2016 [32])	High	High	High	Low	Low
Olsen et al. (2018 [33])	High	High	High	Low	Low
Safi et al. (2021 [34])	High	High	Low	Low	Low
Siyam and Abdallah (2022 [35])	High	High	Low	Low	Low
Sweidan et al. (2019 [36])	High	High	Low	Low	Low
Cabibihan et al. (2017 [37])	High	High	High	Low	Low

Note: Selection bias was high in most studies due to convenience sampling. Performance bias risk was high as blinding of participants and researchers was mostly unreported. Detection bias also posed a potential risk due to unclear blinding of outcome assessors. Attrition bias was low, with most studies reporting low dropout rates. Finally, the lack of preregistered protocols in many studies suggested a possible risk of reporting bias.

## Data Availability

Not applicable.
